# The Role of Sequentially Monitored Laboratory Values and Inflammatory Biomarkers in Assessing the Severity of COVID-19

**DOI:** 10.7759/cureus.51458

**Published:** 2024-01-01

**Authors:** Burcu Baran, Nur A Yetkin, Nuri Tutar, Zeynep Türe, Fatma S Oymak, İnci Gülmez

**Affiliations:** 1 Respiratory Medicine, Erciyes University, Kayseri, TUR; 2 Infectious Diseases, Erciyes University, Kayseri, TUR

**Keywords:** covid-19 pneumonia, laboratory markers, lymphocyte-related biomarkers, crp-related biomarkers, inflammatory biomarkers, covid-19

## Abstract

With the onset of the pandemic in 2020, COVID-19 pneumonia has become a common cause for hospitalization and is associated with high mortality rates. Inflammatory biomarkers play a crucial role in understanding and monitoring the progression of various diseases, including COVID-19. The objective of this study was to assess the significance of sequentially monitored standard laboratory tests, including complete blood cell count, D-dimer, fibrinogen, ferritin, albumin, C-reactive protein (CRP), as well as newly calculated inflammatory biomarkers in predicting the severity and prognosis of COVID-19 pneumonia.

This single-center retrospective study included 194 patients hospitalized due to COVID-19 pneumonia. Patients were grouped based on the severity of their clinical symptoms, with 134 categorized as severe disease and 60 as mild-moderate disease. The patients' demographic data and laboratory values at hospital admission and on the third day of hospitalization were comparatively evaluated.

In the severe illness group, there were more complaints about shortness of breath and a significant drop in the SPO2 value was observed at the time of application (p =0.005 and p<0.001, respectively). The overall mortality rate in all patients was 9% (18/194), and all deaths occurred within the severe disease group. All laboratory parameters, with the exception of platelet count and ferritin levels, were significantly associated and correlated with the severity of the disease during the hospitalization period. Among the biomarkers, there was no significant difference in neutrophil/lymphocyte ratio (NLR) and platelet/lymphocyte ratio (PLR) on the first day, a significant increase was observed on the third day of hospitalization in the severe disease group (p=0.050 vs. 0.003 and p=0.073 vs. 0.020, respectively). No significant difference was observed only in the PNR (platelet/neutrophil ratio) value among the inflammatory biomarkers (p=0.090 vs. p=0.354).

In conclusion, the SPO2 level of COVID-19 patients at admission and the subsequent laboratory parameters examined show a significant relationship with the severity of the disease. In addition, simple inflammation biomarkers derived from laboratory values have shown a very significant relationship and correlation in the diagnosis and follow-up of the disease. In both admission and follow-up evaluation, a more significant association was observed with CRP-related biomarkers such as CRP/albumin ratio and CRP/lymphocyte ratio rather than NLR and PLR, which are widely used in the literature, in showing the severity of COVID-19. In patients with pneumonia, the laboratory assessment made on the third day of hospitalization reflects the severity of the disease more clearly than on the first day.

## Introduction

Coronavirus disease 2019 (COVID-19) caused by a novel coronavirus, which emerged in December 2019, was initially identified in China. This respiratory tract infection exhibits a wide range of symptoms, varying from mild to severe outcomes such as acute respiratory distress syndrome (ARDS) and multiple organ failure [1]. The presence of different clinical manifestations of the disease can be attributed to the genetic variability of severe acute respiratory syndrome coronavirus-2 (SARS-CoV-2), individual characteristics of the patient's immune response to the infection, the initial health status of the patient, and various other factors that influence the pathological process. Patients diagnosed with COVID-19 are categorized based on their clinical presentation, including mild, moderate, severe, and critical cases. The primary factors considered for patient classification are lung involvement and the severity of pneumonia. COVID-19 pneumonia is a frequent reason for hospitalization and is associated with high mortality rates [2].

The complete blood count is a cost-effective, readily available, and efficient method for assessing inflammation in the early detection and prognosis of diseases. The dynamic nature of inflammation in COVID-19 is vital and has a direct correlation with physiological parameters [3]. Inflammatory biomarkers play a crucial role in understanding and monitoring the progression of various diseases, including COVID-19. The COVID-19 pandemic has generated significant interest in identifying biomarkers associated with inflammation, as they can provide valuable insights into disease severity, prognosis, and therapeutic interventions. In severe COVID-19, changes in several parameters of the common blood count have been reported, such as elevated leukocyte and neutrophil counts, increased red cell distribution width (RDW), and persistent decreases in lymphocytes and platelets [3-5].

Recent studies have highlighted the potential utility of novel inflammatory biomarkers, including the neutrophil/lymphocyte ratio (NLR), platelet/lymphocyte ratio (PLR), platelet/ neutrophil ratio (PNR), systemic immune-inflammatory (SII) index, prognostic nutritional index (PNI), C-reactive protein (CRP)/lymphocyte ratio (CLR), CRP/albumin ratio (CAR), and multi-inflammatory index (MII) as valuable indicators for diagnosing and predicting the prognosis of different infectious diseases, including COVID-19 infection [6-8]. These biomarkers are often used as supplementary tools in clinical practice and research to evaluate the inflammatory and immune status of patients and provide insights into prognosis and treatment response. Moreover, it is unclear which inflammation-based index more strongly predicts disease severity in COVID-19 patients.

The objective of this study was to assess the significance of sequentially monitored standard laboratory tests, including complete blood cell count, D-dimer, fibrinogen, ferritin, albumin, CRP, as well as newly calculated inflammatory biomarkers (NLR, PLR, PNR, SII index, PNI, CAR, CLR, MII), in predicting the severity of COVID-19 pneumonia. Furthermore, the correlation between these parameters and disease severity was evaluated by serially measuring them on hospital admission and the third day of hospitalization.

## Materials and methods

Study design and data collection

This single-center retrospective study included 194 patients diagnosed with COVID-19 by SARS-CoV-2 nucleic acid real-time polymerase chain reaction (rt-PCR) testing and hospitalized due to COVID-19 pneumonia, tertiary care hospital in Kayseri, Turkey, between September and December 2021. Patients diagnosed with COVID-19 confirmed by rt-PCR testing, with pneumonia detected on thorax computed tomography (CT), and who were hospitalized and followed up were included in the study. Patients who were under 18 years of age and pregnant were excluded from the study. Demographic data (age, gender, comorbidities), symptoms and clinical findings during hospital admission, laboratory results (at admission and on the third day of hospitalization), length of hospitalization, disease severity, and mortality information were recorded in the study. The hospital has accredited laboratories standardized for internal and external quality assurance measures.

A total of 134 patients with severe disease were included in the study according to the guidelines of the Ministry of Health of Turkey [9]. These patients had to meet at least one of the following criteria: (a) experiencing shortness of breath with a respiratory rate of ≥30 beats/min, (b) having resting oxygen saturation ≤93%, (c) having an arterial oxygen partial pressure (PaO2)/oxygen concentration (FiO2) ≤300 mmHg, or (d) exhibiting lung images showing significant progression of a lesion size >50% within 24 to 48 hours. Patients who required intensive care monitoring due to their clinical conditions (sepsis, myocarditis, etc.) were also included in the severe disease group. The remaining 60 patients, who did not meet these criteria and had a less critical clinical condition, were categorized as having mild to moderate disease.

Ethical approval for this research was obtained from the Ethical Review Board at Erciyes University (No. 2021/215). The study was conducted in accordance with the principles of the Declaration of Helsinki. 

Laboratory

Laboratory test results, including neutrophil count, lymphocyte count, platelet count, CRP, and albumin, were recorded at admission and third day of hospitalization. These results were used to calculate NLR, PLR, PNR, SII index, PNI, CAR, CLR, and MII, which are systemic inflammation biomarkers. NLR was calculated by dividing the neutrophil count by the lymphocyte count, PLR was calculated by dividing the platelet count by the lymphocyte count, PNR was calculated by dividing the platelet count by the neutrophil count and SII index was calculated using the formula: neutrophil count × PLR. The PNI was calculated using the formula: PNI = serum albumin level (g/L) + 5 × total lymphocyte count (/L). CAR was calculated by dividing the CRP level by the albumin level, and CLR was calculated by dividing the CRP level by the lymphocyte count. MII was calculated using the formula NLR × CRP. Additionally, D-dimer, ferritin, and fibrinogen levels at admission and on the third day of hospitalization were also recorded.

Statistical analysis

The SPSS software version 15.0 (IBM Inc., Armonk, New York) was used for statistical analyses of the data. The one-sample Kolmogorov-Smirnov test was used to analyze the distribution of continuous variables; the data were presented as the mean ± SD or median and minimum-maximum ranges. Categorical variables were reported as frequency and group percentages, and the Mann-Whitney U test was used for non-parametric data comparison. Correlations were assessed using Spearman's rank correlation procedure. All p-values less than 0.05 were considered significant.

## Results

A total of 194 patients aged 18 years and older with confirmed COVID-19 pneumonia were retrospectively divided into two groups based on outcomes: 134 patients with severe disease and 60 patients with mild-moderate disease. The median ages (minimum-maximum) were 61 (20-89) and 63 (30-87) years, respectively (p=0.069). Among the total sample, 51% were male (98/194), and there were no significant intergroup differences in terms of sex and age. At least one comorbidity was present in 71% (139/194) of the patients. The most frequently observed comorbidities were diabetes mellitus (n=77, 40%) and hypertension (n=74, 38%). There were no significant differences in comorbidities between the groups (p>0.05 for all).

The most commonly observed symptoms during hospital admission, in order, were fatigue-malaise (n=138, 71%), cough (n=98, 51%), fever (n=87, 45%), and dyspnea (n=84, 43%). The complaint of dyspnea was significantly more common in the severe disease group (p=0.005), while no significant differences were observed between the two groups regarding other symptoms (Table 1).

**Table 1:** A comparative evaluation of the demographic data and hospitalization information of the patients The p*-*value is a comparative statistical evaluation of the parameters of the mild-moderate disease and severe disease groups.

Parameters	Total (n=194)	Mild-moderate disease (n=60)	Severe disease (n=134)	p-value
Female:male ratio, n	96:98	35:25	61:73	0.121
Age in years, median (min-max)	62 (20-89)	61 (20-89)	63 (30-87)	0.069
Comorbidity, n(%)	139 (71)	39 (65)	100 (75)	0.173
Hypertension	74 (38)	18 (30)	56 (42)	0.150
Diabetes mellitus	77 (40)	22 (37)	55 (41)	0.635
Coronary artery disease	36 (19)	10 (17)	26 (19)	0.695
Obstructive pulmonary disease	29 (15)	10 (17)	19 (14)	0.667
Chronic renal failure	25 (13)	6 (10)	19 (14)	0.494
Hyperthyroidism	13 (7)	5 (8)	8 (6)	0.545
Malignancy	22 (11)	6 (10)	16 (12)	0.809
Admission symptoms, n(%)				
Fever	87 (45)	24 (40)	63 (47)	0.435
Dyspnea	84 (43)	17 (28)	67 (50)	0.005
Cough	98 (51)	31 (52)	67 (50)	0.877
Myalgia-arthralgia	50 (26)	19 (32)	31 (23)	0.218
Headache-sore throat	33 (17)	9 (15)	24 (18)	0.684
Fatigue-malaise	138 (71)	40 (67)	98 (73)	0.393
Loss of taste-smell	6 (3)	2 (3)	4 (3)	1.00
Gastrointestinal symptoms	50 (26)	17 (28)	33 (25)	0.598
Admission vital signs, median (min-max)				
SpO2, %	91 (60-100)	94 (91-100)	89 (60-99)	<0.001
Mean Blood Pressure, mmHg	83 (51-110)	83 (70-100)	83 (51-110)	0.873
Pulse rate, beats per minute	86 (56-131)	87 (64-127)	86 (56-131)	0.928
Prevalence of pneumonia infiltration, n(%)				
<50%	101 (52)	39 (65)	63 (47)	0.052
>50%	92 (48)	21 (35)	71 (53)	0.029
Hospitalization, days, median (min-max)	8 (3-38)	6 (3-30)	9 (3-38)	0.001
Oxygen requirement, n(%)	119 (61)	1 (2)	118 (88)	<0.001
Steroid usage, n(%)	141 (73)	37 (62)	104 (78)	0.022
Need for intensive care, n(%)	21 (11)	-	21 (16)	<0.001
Clinical assessment, n(%)				
Mild to moderate pneumonia	60 (31)	60 (31)		
Severe pneumonia	121 (62)		121 (62)	
Acute respiratory distress syndrome (ARDS)	9 (5)		9 (5)	
Septic shock	3 (2)		3 (2)	
Myocarditis	3 (2)		3 (2)	
Mortality, n (%)	18 (9)	-	18 (14)	0.002

In the severe disease group, a significantly lower oxygen saturation (SpO2) value was observed during the first assessment (p<0.001). Furthermore, the radiological assessment revealed a higher prevalence of widespread pneumonic infiltrations (>50%) in the chest CT scans of the severe disease group (p=0.029). The patients in the severe disease group had a longer hospital stay, higher oxygen requirements, increased steroid usage, and a greater need for intensive care compared to those in the mild-moderate disease group (p<0.05 for all).

In the severe disease group, 121 patients developed severe pneumonia, nine patients developed acute respiratory distress syndrome (ARDS), three patients experienced septic shock, and three patients presented with myocarditis. The overall mortality rate in all patients was 9% (18/194), and all deaths occurred within the severe disease group.

In the laboratory examination, as indicated in Table 2, the severe disease group exhibited significantly elevated levels of neutrophil count, D-dimer, fibrinogen, and CRP, as well as reduced levels of albumin both during hospital admission and on the third day of hospitalization (p < 0.05 for all). Although no significant differences were observed in lymphocyte count upon hospital admission, a notable decrease was observed in the severe disease group on the third day of hospitalization (p=0.251 vs. p=0.009). No significant differences were observed between the two groups in terms of platelet count and ferritin levels on hospital admission and the third day of hospitalization (p=0.364 vs. 0.266, and p=0.088 vs. 0.445).

**Table 2:** The comparative evaluation of laboratory values and inflammatory biomarkers during hospital admission and on the third day of hospitalization The data are presented as median (minimum-maximum), as appropriate. The p-value is a comparative statistical evaluation of the parameters of the mild to moderate disease and severe disease groups. CAR - C-reactive protein to albumin ratio; CLR - C-reactive protein to lymphocyte ratio; MII - multi-inflammatory index; NLR - neutrophil-to-lymphocyte ratio; PLR - platelet-to-lymphocyte ratio; PNI - prognostic nutritional index; PNR - platelet-to-neutrophil ratio; SII index - Systemic Immune-Inflammation index

Parameters	Total (n=194)	Mild to moderate disease (n=60)	Severe disease (n=134)	p-value
Hospital admission				
Neutrophil (x10^9^/L)	3.9 (0.9-16.4)	3.6 (1.1-16.4)	4.2 (0.9-16.3)	0.038
Lymphocyte (x10^9^/L)	1.2 (0.2-23)	1.3 (0.2-4.1)	1.2 (0.2-23)	0.251
Platelet (x10^9^/L)	197.5 (12-689)	184 (30-520)	198 (12-689)	0.364
D-dimer (µg /L)	730 (190-80000)	540 (190-80000)	830 (200-18720)	0.001
Fibrinogen (mg/dL)	459 (190-953)	436 (214-733)	471 (190-953)	0.029
Ferritin (ng/mL)	325 (15-9563)	259 (40-4951)	373 (15-9563)	0.088
C-reactive protein (mg/L)	44 (1-347)	31.5 (1-347)	51 (1-271)	0.038
Albumin (g/dL)	3.9 (1.9-4.9)	4.1 (2.4-4.8)	3.8 (1.9-4.9)	0.001
On the third day				
Neutrophil, (x10^9^/L)	5.3 (0.2-22.1)	4.1 (1.3-14.5)	5.9 (0.2-22.1)	0.021
Lymphocyte (x10^9^/L)	0.91 (0-10.8)	1.05 (0.24-4.12)	0.86 (0-10.8)	0.009
Platelet (x10^9^/L)	217.5 (19-705)	205 (110-454)	237 (19-705)	0.266
D-dimer (µg/L)	650 (150-14200)	455 (150-7240)	740 (190-14200)	0.003
Fibrinogen (mg/dL)	484 (122-997)	441 (123-774)	514 (122-997)	0.008
Ferritin (ng/mL)	346.5 (0-7279)	345 (21-2995)	359 (0-7279)	0.445
C-reactive protein (mg/L)	32 (1-243)	20.5 (1-243)	42 (1-222)	0.003
Albumin (g/dL)	3.5 (1.8-4.9)	3.7 (2.2-4.9)	3.5 (1.8-4.3)	0.001
Inflammatory biomarkers				
Hospital admission				
NLR	3.4 (0.1-52.2)	3.2 (0.5-51.5)	3.9 (0.1-52.2)	0.050
PLR	172.3 (1.8-1180)	147.4 (30.9-1180)	175.7 (1.8-835)	0.073
PNR	49 (2.9-223.4)	55.2 (8.4-181.4)	45.6 (2.9-223.4)	0.090
SII index	613.7 (1.6-12154)	510.9 (53.3-12154)	707.6 (1.6-12154)	0.033
PNI	44.5 (3.4-149)	46.8 (4.2-56.5)	43.3 (3.4-149)	0.002
CAR	11.71 (0.22-104.24)	7.88 (0.22-73.83)	13.34 (0.25-104.24)	0.024
CLR	0.0395 (0.0004-0.9394)	0.0227 (0.0005-0.9394)	0.04615 (0.0004-0.5479)	0.042
MII	127.05 (0.3-6574)	80.45 (0.7-4791)	192.80 (0.3-6574)	0.020
On the third day				
NLR	5.6 (0.2-138.4)	4.0 (0.7-26.3)	6.5 (0.2-138.4)	0.003
PLR	214.1 (25.3-1640)	164 (33.5-574.3)	252.2 (25.3-1640)	0.020
PNR	43.2 (0.9-294.2)	46.8 (8.2-134.9)	41.9 (0.9-294.2)	0.354
SII index	1200.2 (88.9-9273.5)	866.8 (100.8-5398.3)	1471.9 (88.9-9273.5)	0.035
PNI	40 (0.7-83)	42.3 (7.5-55.5)	39.3 (0.7-83)	0.016
CAR	8.89 (0.22-00.91)	5.47 (0.22-65.14)	12.64 (0.25-100.91)	0.002
CLR	0.0377 (0.0001-1.1200)	0.0224 (0.0004-0.3471)	0.0426 (0.0001-1.1200)	0.001
MII	147.9 (0.4-10550.8)	90 (0.8-2861.3)	188 (0.4-10550.8)	0.001

Similarly, in both hospital admission and on the third day of hospitalization, the inflammatory biomarkers SII index, CAR, CLR, and MII, were found to be elevated in the severe disease group, while PNI showed a significant decrease (p=0.033 vs. 0.035, p=0.024 vs. 0.002, p=0.042 vs. 0.001, p=0.020 vs. 0.001, and p= 0.002 vs. 0.016, respectively; Table 2). While there was no significant difference in NLR and PLR on the first day, a significant increase was observed on the third day of hospitalization in the severe disease group (p=0.050 vs. 0.003 and p=0.073 vs. 0.020, respectively). No significant difference was observed only in the PNR value among the inflammatory biomarkers (p=0.090 vs. 0.354).

The correlation between disease severity and hospitalization duration, admission SPO2 value, and the laboratory values were evaluated. There was no significant correlation observed between platelet count, ferritin, and PNR values and disease severity (p=0.366 vs. 0.268, p=0.088 vs. 0.447, and p=0.090 vs. 0.357, respectively). D-dimer, albumin, and PNI values measured at hospitalization and on the third day of hospitalization showed a similarly significant correlation with disease severity (p=0.001 vs. 0.003, p=0.001 vs. 0.001, and p=0.002 vs. 0.016, respectively). However, laboratory parameters such as neutrophil count, lymphocyte count, CRP, fibrinogen, and inflammatory biomarkers such as NLR, PLR, SII index, CAR, CLR, and MII measured on the third day of hospitalization showed a stronger and more significant correlation with disease severity (Table 3). According to all parameters, the most significant correlation with disease severity was observed with the admission SPO2 value (r=-652, p<0.001). 

**Table 3:** Correlation between disease severity and clinical/laboratory parameters CRP - C-reactive protein; CAR - C-reactive protein to albumin ratio; CLR - C-reactive protein to lymphocyte ratio; MII - multi-inflammatory index; NLR - neutrophil-to-lymphocyte ratio; PLR - platelet-to-lymphocyte ratio; PNI - prognostic nutritional index; PNR - platelet-to-neutrophil ratio; SII index - Systemic Immune-Inflammation Index

Parameters	First day r	First day p	Third day r	Third day p
Admission SPO2	-0.652	<0.001	NA	NA
Hospitalization time	0.249	0.001	NA	NA
Neutrophil count	0.149	0.038	0.167	0.021
Lymphocyte count	-0.083	0.252	-0.19	0.008
Platelet count	0.065	0.366	0.113	0.268
D-dimer	0.255	0.001	0.242	0.003
Fibrinogen	0.175	0.029	0.23	0.007
Ferritin	0.134	0.088	0.064	0.447
Albumin	-0.244	0.001	-0.253	0.001
NLR	0.141	0.05	0.212	0.003
PLR	0.129	0.072	0.238	0.019
PNR	-0.122	0.09	-0.095	0.357
SII index	0.154	0.034	0.216	0.032
PNI	-0.222	0.002	-0.175	0.016
CRP	0.149	0.038	0.215	0.003
CAR	-0.166	0.023	-0.229	0.002
CLR	0.147	0.041	0.239	0.001
MII	0.168	0.019	0.25	0.001

## Discussion

The findings of this study provide supporting evidence in line with existing guidelines, suggesting that the presence of dyspnea and low SPO2 levels at admission, as well as the presence of extensive pneumonia observed on thorax CT scans, are associated with severe disease in patients diagnosed with COVID-19 pneumonia [2,10]. In a meta-analysis that included the evaluation of 69 studies [11], a significant association was found between the symptoms of dyspnea and mortality. Similarly, in our study, we observed a significant increase in dyspnea as a presenting symptom among patients with severe disease (p=0.005). While advanced age, male gender, and the presence of hypertension, diabetes, and cardiovascular comorbidities are known risk factors for severe disease and mortality in COVID-19 [10-13]. Incompatible with the literature, no significant differences were observed between the two groups in terms of gender, age, and comorbidities in this study. The SPO2 level during hospital admission was also associated with severe illness, similar to Khadzhieva et al.'s study (90% (87-95%) vs. 94% (91-96%), p=0.0278) and (89% (60-99%) vs. 94% (91-100%), p<0.001) [3]. As expected, hospitalization duration, steroid usage, oxygen requirement, and intensive care need significantly increased in the severe disease group. It is noteworthy that the overall mortality rate among all patients was 9% (18/194), with all fatalities occurring within the severe disease group.

We evaluated the sequential changes in various laboratory parameters and inflammatory biomarkers. In the severe disease group, the neutrophil count, D-dimer, fibrinogen, and CRP exhibited significant elevation compared to the initial assessment, whereas the albumin level showed a significant decrease. These significant differences in laboratory values persisted on the third day of hospitalization as well. However, no significant association was observed between disease severity and platelet count or ferritin levels. The lymphocyte count significantly decreased in the severe disease group on the third day of hospitalization (p=0.251 vs. p=0.009). Notably, the inflammatory biomarkers SII index, PNI, CAR, CLR, and MII demonstrated a significant association with disease severity during both admission and follow-up.

The number of neutrophils secreting pro-inflammatory cytokines increases in patients with COVID-19, particularly in severe and critical course of the disease. This condition is caused by a strong activation of the non-specific cellular response and promotes the development of lymphopenia. NLR indicates the correlation between these two populations, potentially serving as an early predictor of the disease's severe progression. Many studies in the literature have shown that the NLR parameter is a highly significant biomarker in predicting COVID-19 disease severity (AUC>0.91). However, differently, in some studies, no statistically significant difference was found between COVID-19 disease severity and mortality and NLR values [14-19]. In this study, consistent with the literature, neutrophil count was significantly increased at hospital admission, and no significant difference was found in lymphocyte count and NLR levels. However, neutrophilia, lymphopenia, and increased NLR became significantly more pronounced with disease progression on the third day of hospitalization.

Platelets play an important role in blood clotting and the immune response to infections. Studies have shown that platelet counts are reduced in COVID-19 patients and are associated with disease severity [14-19]. In our study, a a significant increase in neutrophil count was observed, while no difference was observed in lymphocyte and platelet counts. However, on the third day, all parameters except platelet count showed a significant difference between the two groups. It was confirmed that PLR and PNR values reached higher levels in severe or critically ill patients than in those with milder COVID-19 infection. In this study, NLR, PLR, and PNR values did not differ significantly at the time of diagnosis, but NLR and PLR increased significantly in the severe disease group during the follow-up period (p=0.050 vs. p=0.003 and p=0.073 vs. p=0.020, respectively), while PNR did not make this significant difference (p=0.090 vs. p=0.354). Among these parameters, we can say that lymphopenia has the clearest relationship with disease severity. 

Ferritin is a protein responsible for iron storage in the body and can also increase during inflammatory conditions. Research conducted during the COVID-19 pandemic has shown a relationship between ferritin levels and the severity and prognosis of the disease. Some studies suggest that elevated ferritin levels in COVID-19 patients may increase the likelihood of severe disease and contribute to the development of critical conditions [17,20]. In our study, ferritin levels were not found to be associated with the severity of the disease, both at the time of hospital admission and during follow-up (p=0.088 vs. p=0.445, respectively).

The SII index is calculated by multiplying the platelet count by the NLR and has been proposed as a marker of systemic inflammation and immune response. Some studies have suggested that a higher SII index is associated with increased disease severity and worse outcomes in COVID-19 patients. Elevated SII levels have been linked to a more intense inflammatory response and coagulopathy, which may contribute to the development of severe complications such as acute respiratory distress syndrome (ARDS) and thromboembolic events [21,22]. The study conducted by Gujar et al. demonstrated that the SII parameter had a high diagnostic value (AUC=0.841) in distinguishing the severity of COVID-19. Critically ill patients admitted to the ICU showed a significantly higher mean SII value compared to the control group in-home or hospital isolation (mean SII value: 2484 vs. 653.3) (p=0.001) [21]. Hamad et al. also found a high diagnostic value for SII, with an AUC of 0.819. Critically ill patients in the ICU had a mean SII value of 2016.29, whereas patients not requiring ICU hospitalization had a considerably lower mean SII value of 492.29. The diagnostic sensitivity of SII was 50.9%, and the specificity was 95.6% in predicting the risk of deterioration and ICU hospitalization [22]. Similarly, in this study, the SII index exhibited a significant increase in the group of patients with severe disease both at the time of hospital admission and during follow-up (at admission 707.6 vs. 510.9, p=0.033; third day of hospitalization 1471.9 vs. 866.8, p=0.035), indicating a positive correlation.

Accumulating evidence suggests that severe systemic inflammation and poor nutritional status are associated with a poorer prognosis in patients with COVID-19. PNI, which incorporates lymphocyte and albumin levels, is an effective index for assessing inflammation and nutritional status. Wang et al. demonstrated that a low PNI at admission is an independent factor for mortality in COVID-19 patients [23]. Similarly, Aciksari et al. reported an association between PNI≤40.2 and increased mortality, with an odds ratio of 10.85 [24]. Çakirca et al. found that a PNI≤40.03 was an independent predictor of mortality in COVID-19 patients and highlighted that PNI had the highest AUC among inflammation-based indices for predicting mortality [25]. Consistent with these findings, our study revealed significantly lower PNI values in the severe disease group at both admission and on the third day (p = 0.002 and p = 0.016, respectively). Furthermore, the median PNI value on the third day in the severe disease group was 39.3 (0.7-83), supporting the previously established cutoff value. Additionally, there was a significant negative correlation between PNI values and disease severity at both admission and on the third day (r=-0.222, p=0.002 vs. r=-0.175, p=0.016).

Of late, using CAR as an indicator has been regarded as effective in prognosticating outcomes in conditions such as cancer, sepsis, and critical illnesses [26,27]. Ertekin et al. evaluated the significance of albumin levels and their proportion to other biomarkers in predicting mortality among severe COVID-19 patients; the CRP and CAR levels of the mortality group were found to be higher than those who survived (p<0.001). Furthermore, the AUC of CAR was higher than that of CRP and albumin (AUC 0.806, 0.745, 0.772, respectively). When the CAR cutoff value was >3.7, it had 79.6% sensitivity, 65.4% specificity, and p<0.001 [28]. Karakoyun et al. stated that CAR may have a major role in systemic inflammation and can predict the severity of COVID-19 in earlier stages compared to CRP and albumin [29]. In this study, as well, the laboratory parameters that exhibited the most significant relationship and correlation with disease severity during hospital admission and follow-ups were CRP, albumin, and the calculated CAR and CLR parameters. 

The MII is the product of NLR and CRP, which have been considered biomarkers of lethal outcomes in COVID-19. In the context of the development and severity of COVID-19, MII demonstrated the best performance for predicting mortality among all inflammatory markers studied in the study, with no differences in PLR and SII found between survivors and non-survivors [30]. Khadzhieva et al. conducted an assessment of sequential laboratory markers linked to CRP in COVID-19 patients. The study investigated the correlation between these markers and patient prognosis over a period spanning from day one to day 30. Notably, upon admission, individuals who did not survive exhibited elevated CLR and MII values compared to those who survived. However, at the point of discharge or death, the most notable distinctions were observed concerning the NLR, Systemic Inflammation Response Index (SIRI), and MII [3]. Consistent with the literature, in this study, the MII value was significantly higher upon admission and on the third day of hospitalization in the severe disease group (p=0.020 and p=0.001, respectively). In addition, it demonstrated a significant positive correlation with disease severity during both hospital admission and follow-up (r=0.168, p=0.019 and r=0.250, p=0.001).

The most significant correlation with disease severity among all laboratory parameters at admission was observed with D-dimer and albumin values (r=0.255, p=0.001, and r=-0.253, p=0.001). While stronger correlations were observed, particularly in the values on the third day, for the laboratory parameters and biomarkers, no correlation was found for platelet count, ferritin, and PNR values (p>0.05). The most significant correlation with disease severity was observed with the admission SPO2 value (r=-652, p<0.001). 

Our study has several limitations, including a retrospective design and a relatively small sample size. Therefore, these findings warrant confirmation in other populations, particularly by multicenter studies. We studied the sequential changes in the values of complete blood cell count, D-dimer, fibrinogen, ferritin, albumin, CRP, as well as newly calculated inflammatory biomarkers such as NLR, PLR, PNR, SII index, PNI, CAR, CLR, and MII. In both admission and follow-up evaluation, a more significant association was observed with CRP-related inflammatory biomarkers such as CRP/albumin ratio and CRP/lymphocyte ratio rather than NLR and PLR, which are widely used in the literature, in showing the severity of COVID-19.

## Conclusions

In conclusion, the utilization of this series of laboratory values and inflammatory biomarkers is important for categorizing patients, identifying high-risk groups, and monitoring the treatment process of COVID-19 patients. It is necessary to develop customized prognostic models that can adapt to specific circumstances, allowing for continuous monitoring and potential adjustments as needed. The identification of novel markers that exhibit greater sensitivity in the early stages of the disease, along with the development of prognostic tools based on biomarkers, has the potential to significantly enhance the outcomes of COVID-19.
